# MiRNA-423 rs6505162 and miRNA-6811 rs2292879 SNP associated with lung cancer in Hainan, China

**DOI:** 10.1042/BSR20231152

**Published:** 2023-09-22

**Authors:** Jing Zhou, Chong Meng, Yixuan Li, Yihui Fu, Wenfang Long, Hairong Huang, Yunru Liu, Pengfei Lyu, Sha Xiao

**Affiliations:** 1 International School of Public Health and One Health, Laboratory of Tropical Environment and Health, Heinz Mehlhorn Academician Workstation, Hainan Medical University, Haikou 571199, China; 2 Department of Respiratory and Critical Care Medicine, Hainan General Hospital, Hainan Affiliated Hospital of Hainan Medical University, Haikou 570311, China; 3 Department of Breast Surgery, the First Affiliated Hospital of Hainan Medical University, Haikou, Hainan 570102, China

**Keywords:** genetic susceptibility, Lung cancer, microRNA genes, single nucleotide polymorphisms

## Abstract

Background: MicroRNAs (miRNAs) are known to exert significant influence on various physiological processes and diseases, including cancers. The primary objective of this present study was to examine the impact of eight single-nucleotide polymorphisms (SNPs) in miRNA on the susceptibility to lung cancer (LC) within the Chinese Southern population.

Methods: The genotypes of these eight polymorphisms were determined in 132 LC patients and 214 cancer-free controls.

Results: In overall analyses, GG genotype of miRNA-6811 rs2292879 polymorphism was significantly correlated with increased risk of LC (GG vs. AA, adjusted OR = 5.10, 95% CI = 1.02–25.43, *P*=0.047), yet the genotype frequencies of rs2292879 SNP in controls did not met the Hardy–Weinberg equilibrium (HWE) (*P*=0.001) in present study. Stratified analyses by smoking revealed that miRNA-423 rs6505162 variants significantly decreased the LC risk in heterozygous (CA vs. CC, adjusted OR = 0.14, 95% CI = 0.03–0.81, *P*=0.028) and recessive (AA vs. CA + CC, adjusted OR = 0.17, 95% CI = 0.03–0.90, *P*=0.038) genetic models in smoking population. However, miRNA-196A2 rs11614913, miRNA-196A2 rs12304647, miRNA-146A rs2910164, miRNA-16-1 rs1022960, miRNA-608 rs4919510, and miRNA-27a rs895819 polymorphisms were not significantly associated with LC.

Conclusion: The findings of our study indicate a potential decrease in LC risk among smokers with the miRNA-423 rs6505162 variants, while an increase in risk is associated with miRNA-6811 rs2292879 polymorphisms in the population of Southern Chinese. However, further well-designed research is necessary to fully understand the precise impact of these two SNPs on the development of LC.

## Introduction

Lung cancer (LC) has been of great concern due to its high prevalence and mortality worldwide. There were more than 2.2 million newly diagnosed LC cases and nearly 1.8 million LC-related deaths, remaining the leading cause of cancer-associated death around the world in 2020 [1]. In China, there were approximately 0.82 million new LC cases and 0.71 million LC-related deaths, still being one of the major public health issues in 2020 [1]. Diagnosis of LC is often delayed because early stage disease is typically asymptomatic, resulting in most LC patients being advanced at diagnosed. It was reported that only 30% of cases were diagnosed at stage I, resulting in a five-year survival rate of 65%, which significantly drops to 5% or 6% for patients with advanced LC [2,3]. Thus, the earlier diagnosis is of great importance in the prognosis of LC patients and searching for specific and sensitive diagnostic molecular markers for early diagnosis of LC is significant.

Emerging evidence suggests a substantial role for microRNAs (miRNAs) in the LC development and progression [4–8]. MiRNA is a kind of endogenous non-coding small RNA (ncRNA), which regulates its target gene expression as a part of the RNA-induced silencing complex (RISC) [9,10]. It was estimated that approximately 30% of the human coding protein genes are regulated by miRNAs [11]. The mechanisms of miRNA-mediated gene expression include translational repression, mRNA cleavage, post-transcriptional regulation through binding to 3′-untranslated regions (3′-UTRs) of target RNAs [9,12,13]. The aberrant expression or dysfunction of miRNAs has been extensively documented to be associated with the pathogenesis and advancement of numerous human diseases, such as diabetes [14], depressive disorder [15], rheumatoid arthritis [16], among others. Of particular significance is the involvement of miRNAs in cancer, with multiple miRNAs identified as either tumor suppressors or tumor promoters [4,17,18]. In the context of cancer, miRNAs have the potential to modulate various cellular processes, including cell proliferation, invasion, metastasis, angiogenesis, and immune evasion [9,18,19]. Hence, a multitude of miRNAs have been suggested as potential biomarkers for cancer diagnosis, treatment, and prognosis [20,21].

LC is commonly acknowledged to be influenced by a combination of genetic and environmental factors, as well as interactions between genes and the environment [22]. Genetically distinct individuals experiencing the same environmental exposures may vary in their susceptibility to LC [22]. Extensive research has shown that SNPs in miRNA can contribute to alteration in miRNA-mediated gene expression by affecting the transcription, processing, and maturation of pri-miRNA and pre-miRNA, as well as miRNA targeting, which might implicate in cancer initiation and development [23–25]. The present study aimed to evaluate the association of SNPs (miRNA-423 rs6505162, miRNA-6811 rs2292879, miRNA-16-1 rs1022960, miRNA-608 rs4919510, miRNA-146A rs2910164, miRNA-196A2 rs11614913, miRNA-196A2 rs12304647, and miRNA-27a rs895819) in miRNAs with LC genetic susceptibility in a Southern Chinese population.

## Materials and methods

### Study subjects

We recruited 132 patients, whom were histopathologically confirmed as new lung cancer cases and had never received any radiotherapy or chemotherapy before diagnosis, from two hospitals (Hainan General Hospital and the First Affiliated Hospital of Hainan Medical University) from January 2017 through October 2017. During the same period, 214 controls with no evidence of any cancer were enrolled from medical examination center in the same hospital. Participants were all genetically unrelated Chinese mainly living in Hainan Province of China. This study obtained approval from the Research Ethics Committee of Hainan Medical University in compliance with the Helsinki Declaration (HYLL-2019-034). Prior to conducting human investigation and collecting blood samples, written informed consent was obtained from each participant or their representatives in cases where direct consent was not available.

Each participant provided a 2 ml of sample of peripheral blood before treatment and completed a questionnaire to collect the demographic data.

### SNP selection

Based on previous studies, SNPs with a minor allele frequency (MAF) exceeding 5% in East Asian population enrolled in 1000 Genomes Project were identified using the Ensembl browser (http://feb2023.archive.ensembl.org) and dbSNP database (http://www.ncbi.nlm.nih.gov/snp). Ultimately, eight SNPs (miRNA-423 rs6505162, miRNA-6811 rs2292879, miRNA-16-1 rs1022960, miRNA-608 rs4919510, miRNA-146A rs2910164, miRNA-196A2 rs11614913, miRNA-196A2 rs12304647, and miRNA-27a rs895819) were selected as candidate SNPs for further evaluation.

### DNA extraction and SNP genotyping

Genomic DNA was extracted from the blood samples using the TaKaRa DNA Extraction Kit (TaKaRa, Dalian, China), following the manufacturer’s protocol meticulously. Purity and concentration of the DNA samples were assessed, and subsequently, the genotypes of these eight SNPs were detected applying the Applied Biosystems 3730XL DNA Analyzer (ThermoFisher Scientific, Waltham, MA, U.S.A.).

### Statistical methods

Differences in the distribution of demographic and lifestyle characteristics between cases and controls were evaluated by the Pearson Chi-square test or Fisher’s exact test. The observed and expected genotype frequencies in controls were compared by Chi-square test in order to assess HWE. Logistic regression analysis with and without adjustment for potential confounders (age, gender, family history of tumor, smoking, and/or alcohol consumption), was performed to calculating odds ratios (ORs) and 95% confidence intervals (CIs) for evaluating the associations between SNPs and LC risks. All the statistical analyses were conducted using SPSS v20.0 (IBM Analytics, Chicago, IL, U.S.A.), and two-sided *P*-value < 0.05 was considered statistical significance.

## Results

### Characteristics of cases and controls

In total 346 subjects including 132 patients with LC (42 females and 90 males) and 214 cancer-free controls (84 females and 130 males) were enrolled in the present study. The participants’ characteristics were summarized in Table 1. There were no statistically significant differences observed in the distribution of age, gender, or family history of tumor between the case and control groups (*P*=0.212, 0.163, 1.000, respectively). However, the rates of smoking and alcohol consumption were both higher in patients than in the cancer-free controls (*P*=0.004 and 0.009, respectively). Among the 132 cases of LC, adenocarcinoma accounted for 69 (52.3%), squamous cell carcinoma for 29 (22.0%), small cell carcinoma for 22 (16.7%), and other types for 12 (9.1%).

**Table 1:** Demographic, lifestyle characteristics, and selected LC complication risk factors of study population

Characteristics	Control (*N*=214)	Case (*N*=132)	*P*
Age, year, *n* (%)			0.212
<50	37 (17.3)	29 (22.0)	
50–60	46 (21.5)	35 (26.5)	
≥60	131 (61.2)	68 (51.5)	
Gender, *n* (%)			0.163
Women	84 (39.3)	42 (31.8)	
Men	130 (60.7)	90 (68.2)	
Smoking, *n* (%)^*^			**0.004**
No	123 (57.5)	55 (41.7)	
Yes	91 (42.5)	77 (58.3)	
Alcohol drinking, *n* (%)^†^			**0.009**
No	179 (83.6)	95 (72.0)	
Yes	35 (16.4)	37 (28.0)	
Family history of cancer, *n* (%)			1.000
No	210 (98.1)	130 (98.5)	
Yes	4 (1.9)	2 (1.5)	
Histology, *n* (%)			
Adenocarcinoma	–	69 (52.3)	
Squamous cell carcinoma	–	29 (22.0)	
Small cell carcinoma	–	22 (16.7)	
Others	–	12 (9.1)	
Stage, *n* (%)			
I –II	–	14 (10.6)	
III –IV	–	118 (89.4)	

* Smoking was defined as having smoked at least one cigarette daily for at least six consecutive months at any time during the person’s life.

† Alcohol drinking was defined as consuming an alcoholic drink at least once per week for at least six consecutive months at any time during the person’s life.

### Information about the candidate SNPs

Details of the eight targeted SNPs were shown in Table 2. MAFs of all SNPs were above 5% in the East Asian population, and the genotype frequencies of the eight SNPs were all in HWE for the control group (*P*>0.05), except for miRNA-16-1 rs1022960 and miRNA-6811 rs2292879.

**Table 2:** Information about the eight selected SNPs in miRNAs

miRNAs	SNP ID	Base change	MAF			*P*-value for HWE
			Control	Case	Reference^*^	
miRNA-423	rs6505162	C>A	0.24	0.27	0.23	0.835
miRNA-6811	rs2292879	A>G	0.22	0.24	0.34	**0.001**
miRNA-16-1	rs1022960	C>T	0.43	0.41	0.35	**0.045**
miRNA-608	rs4919510	G>C	0.47	0.46	0.41	0.152
miRNA-146A	rs2910164	C>G	0.38	0.43	0.37	0.588
miRNA-196A2	rs11614913	T>C	0.43	0.46	0.49	0.149
miRNA-196A2	rs12304647	A>C	0.23	0.22	0.28	0.194
miRNA-27a	rs895819	T>C	0.23	0.24	0.26	0.055

* MAF from 1000 Genomes Project (http://feb2023.archive.ensembl.org) for population of East Asian.

### Model of inheritance analysis of the eight SNPs with LC

Table 3 shows the association between the eight SNPs and the risk for LC. For miRNA-423 rs6505162, the results of crude ORs (ORs were 0.34 and 0.29, 95% CIs were 0.12–0.94, and 0.10−0.85, *P-*values were 0.038 and 0.023, respectively) indicated that individuals carrying heterozygous CA or homozygous AA genotype had lower risks of LC compared with the wild homozygous CC genotype. In the recessive model, we found that rs6505162 homozygous AA of miRNA-423 were associated with a significantly decreased risk of LC compared with its heterozygous CA and wild-type homozygote CC (crude OR = 0.32, 95% CI = 0.11−0.88, *P*=0.027). However, the aforementioned associations appeared to be untrue based on the results of adjusted ORs. As for miRNA-6811 rs2292879 polymorphism, individuals with GG genotype were found to have a higher likelihood of developing LC when compared with those with the AA genotype (adjusted OR = 5.10, 95% CI = 1.02−25.43, *P*=0.047). However, it is important to note that this finding should be verified through extensive and well-designed studies, as the distribution of SNP rs2292879 in the control group did not align with the HWE (*P*=0.001) in the present study. Our study did not find any significant associations between the rs1022960, rs4919510, rs2910164, rs11614913, rs12304647, or rs895819 polymorphisms and LC.

**Table 3:** Genotype frequencies of the eight miRNA-SNPs in patients and controls and associations between them and the risk of LC

SNP/Genotype/Genetic model	*N* (%)		OR (95%CI)	*P*	OR_adj_ (95%CI)^*^	*P*
	Case (132)	Control (214)				
miRNA 423 rs6505162						
CC	73 (55.3)	119 (55.6)	1.00 (ref.)			
CA	48 (36.4)	89 (41.6)	0.34 (0.12–0.94)	**0.038**	0.39 (0.13–1.15)	0.089
AA	11 (8.3)	6 (2.8)	0.29 (0.10–0.85)	**0.023**	0.34 (0.11–1.00)	0.050
Dominant model CA+AA vs. CC			0.99 (0.64–1.53)	0.956	1.04 (0.66–1.63)	0.877
Recessive model AA vs. CA+CC			0.32 (0.11–0.88)	**0.027**	0.37 (0.13–1.06)	0.063
Allele model A vs.C			0.86 (0.06–1.22)	0.388	0.90 (0.62–1.29)	0.550
miRNA 6811 rs2292879						
AA	70 (53.0)	129 (60.3)	1.00 (ref.)			
AG	60 (45.5)	76 (35.5)	2.44 (0.51–11.62)	0.262	3.42 (0.69–16.90)	0.131
GG	2 (1.5)	9 (4.2)	3.55 (0.74–17.06)	0.113	5.10 (1.02–25.43)	**0.047**
Dominant model AG+GG vs. AA			0.74 (0.48–1.15)	0.186	0.75 (0.48–1.18)	0.210
Recessive model GG vs. AG+AA			2.85 (0.61–13.42)	0.184	4.04 (0.83–19.70)	0.084
Allele model G vs. A			0.88 (0.61–1.26)	0.488	0.92 (0.63–1.34)	0.656
miRNA 16-1 rs1022960			0.82 (0.60–1.12)	0.218	0.84 (0.61–1.16)	0.289
CC	45 (34.1)	64 (29.9)	1.00 (ref.)			
CT	67 (50.8)	116 (54.2)	1.20 (0.61–2.34)	0.602	1.48 (0.73–2.99)	0.279
TT	20 (15.1)	34 (15.9)	0.98 (0.52–1.84)	0.995	1.26 (0.65–2.44)	0.500
Dominant model CT+TT vs. CC			1.21 (0.76–1.93)	0.416	1.24 (0.77–2.00)	0.385
Recessive model TT vs. CT+CC			1.06 (0.58–1.93)	0.855	1.33 (0.71–2.52)	0.370
Allele model T vs. C			1.11 (0.81–1.51)	0.524	1.18 (0.85–1.63)	0.316
miRNA 608 rs4919510						
GG	33 (25.0)	50 (23.4)	1.00 (ref.)			
GC	78 (59.1)	127 (59.3)	1.16 (0.58–2.33)	0.669	1.31 (0.64–2.68)	0.459
CC	21 (15.9)	37 (17.3)	1.08 (0.59–1.98)	0.798	1.10 (0.59–2.05)	0.768
Dominant model GC+CC vs. GG			1.09 (0.66–1.81)	0.729	1.22 (0.72–2.05)	0.460
Recessive model CC vs. GC+GG			1.11 (0.62–1.99)	0.738	1.16 (0.63–2.12)	0.638
Allele model C vs. G			1.06 (0.78–1.45)	0.699	1.12 (0.82–1.54)	0.481
miRNA 146A rs2910164						
CC	35 (26.5)	69 (32.2)	1.00 (ref.)			
CG	81 (61.4)	127 (59.3)	0.57 (0.26–1.25)	0.162	0.57 (0.25–1.28)	0.175
GG	16 (12.1)	18 (8.4)	0.72 (0.37–1.49)	0.372	0.66 (0.31–1.40)	0.281
Dominant model CG+GG vs. CC			0.76 (0.47–1.23)	0.260	0.81 (0.50–1.33)	0.412
Recessive model GG vs. CG+CC			0.67 (0.33–1.36)	0.263	0.63 (0.30–1.31)	0.217
Allele model G vs. C			0.92 (0.59–1.43)	0.710	1.00 (0.63–1.58)	0.989
miRNA 196A2 rs11614913						
CC	38 (28.8)	72 (33.6)	1.00 (ref.)			
CT	65 (49.2)	102 (47.7)	0.73 (0.39–1.35)	0.215	0.76 (0.10–1.44)	0.399
TT	29 (22.0)	40 (18.7)	0.88 (0.50–1.56)	0.658	0.91 (0.50–1.64)	0.746
Dominant model CT+CC vs. TT			0.80 (0.50–1.28)	0.346	0.81 (0.50–1.32)	0.406
Recessive model CC vs. CT+TT			0.82 (0.48–1.40)	0.459	0.85 (0.49–1.48)	0.557
Allele model T vs. C			0.85 (0.62–1.15)	0.295	0.86 (0.63–1.18)	0.353
miRNA 196A2 rs12304647						
AA	78 (59.1)	125 (58.4)	1.00 (ref.)			
AC	51 (38.6)	78 (36.5)	2.29 (0.62–8.46)	0.215	2.85 (0.74–10.98)	0.127
CC	3 (2.3)	11 (5.1)	2.40 (0.64–9.02)	0.196	2.72 (0.70–10.66)	0.150
Dominant model AC+CC vs. AA			1.03 (0.66–1.60)	0.901	1.14 (0.72–1.81)	0.570
Recessive model CC vs. AC+AA			2.33 (0.64–8.51)	0.201	2.80 (0.74–10.63)	0.130
Allele model C vs. A			1.11 (0.77–1.60)	0.589	1.20 (0.82–1.76)	0.342
miRNA 27a rs895819						
TT	78 (59.1)	126 (58.9)	1.00 (ref.)			
TC	46 (34.8)	78 (36.4)	0.77 (0.29–2.05)	0.605	0.87 (0.34–2.41)	0.787
CC	8 (6.1)	10 (4.7)	0.74 (0.27–2.00)	0.549	0.84 (0.30–2.40)	0.749
Dominant model TC+CC vs. TT			1.01 (0.65–1.57)	0.969	1.01 (0.64–1.59)	0.970
Recessive model CC vs. TC+TT			0.76 (0.29–1.98)	0.573	0.86 (0.32–2.34)	0.767
Allele model C vs. T			0.97 (0.67–1.39)	0.859	0.98 (0.68–1.43)	0.933

* OR_adj_ and *P*-value were calculated and adjusted for age, gender, smoking, alcohol consumption, and family history of tumor by a logistic regression model.

### Stratified analysis of the eight SNPs with LC

Further analyses were performed stratified by either smoking or alcohol consumption (Tables 4 and 5). Results revealed that miRNA-423 rs6505162 (C>A) polymorphism may be associated with reduced susceptibility of LC in the group of smokers. Comparing with rs6505162 CC homozygote among smokers, CA heterozygote was associated with a significantly decreased LC risk (adjusted OR = 0.14, 95% CI = 0.03–0.81, *P*=0.028) and smoking individuals with rs6505162 AA genotype also had a reduced susceptibility to LC (Recessive model, adjusted OR = 0.17, 95% CI = 0.03–0.90, *P*=0.038). However, no significant associations were observed between the other seven variants (rs11614913, rs12304647, rs2910164, rs1022960, rs4919510, rs895819, and rs2292879) and LC risk within smoking subpopulation. Moreover, we did not find any main effects between these eight selected SNPs and LC risk in non-smokers, alcohol drinkers, or non-alcohol drinkers.

**Table 4:** Associations between the eight miRNA-SNPs and the risk of LC stratified by smoking

SNP/Genotype/Genetic model	Smoking				Non-smoking			
	OR (95%CI)	*P*	OR_adj_ (95%CI)^*^	*P*	OR (95%CI)	*P*	OR_adj_ (95%CI)^*^	*P*
miRNA 423 rs6505162								
CC	1.00 (ref.)				1.00 (ref.)			
CA	0.14 (0.03–0.78)	**0.025**	0.14 (0.03–0.81)	**0.028**	0.57 (0.09–3.64)	0.551	0.62 (0.09–4.24)	0.623
AA	0.20 (0.04–1.11)	0.065	0.20 (0.04–1.13)	0.068	0.35 (0.05–2.34)	0.277	0.37 (0.05–2.62)	0.319
Dominant model CA+AA vs. CC	0.61 (0.32–1.17)	0.135	0.63 (0.33–1.20)	0.157	1.44 (0.65–3.18)	0.368	1.50 (0.66–3.39)	0.336
Recessive model AA vs. CA+CC	0.17 (0.03–0.88)	**0.035**	0.17 (0.03–0.90)	**0.038**	0.48 (0.08–2.97)	0.425	0.50 (0.07–3.32)	0.470
Allele model A vs. C	1.21 (0.74–1.98)	0.457	0.61 (0.36–1.02)	0.057	0.60 (0.36–1.00)	0.048	1.31 (0.78–2.18)	0.311
miRNA 6811 rs2292879								
AA	1.00 (ref.)				1.00 (ref.)			
AG	0.35 (0.02–5.86)	0.469	0.49 (0.03–8.31)	0.620	5.59 (0.63–49.46)	0.122	7.01 (0.76–65.00)	0.087
GG	0.61 (0.04–10.09)	0.726	0.86 (0.05–14.81)	0.916	3.92 (0.43–35.53)	0.224	4.93 (0.51–47.59)	0.168
Dominant model AG+GG vs. AA	0.58 (0.30–1.10)	0.095	0.57 (0.29–1.09)	0.090	1.66 (0.76–3.61)	0.205	1.73 (0.77–3.88)	0.182
Recessive model GG vs. AG+AA	0.44 (0.03–7.21)	0.567	0.61 (0.04–10.22)	0.730	4.80 (0.56–41.36)	1.053	6.15 (0.68–55.83)	0.106
Allele model G vs. A	1.21 (0.74–1.98)	0.457	0.66 (0.38–1.13)	0.128	0.66 (0.38–1.12)	0.123	1.23 (0.74–2.05)	0.422
miRNA 16-1 rs1022960								
CC	1.00 (ref.)				1.00 (ref.)			
CT	1.49 (0.50–4.45)	0.475	1.53 (0.50–4.70)	0.454	0.87 (0.28–2.73)	0.810	0.99 (0.29–3.39)	0.983
TT	1.10 (0.39–3.07)	0.862	1.13 (0.40–3.22)	0.815	0.75 (0.25–2.27)	0.611	0.85 (0.26–2.76)	0.789
Dominant model CT+TT vs. CC	1.38 (0.70–2.73)	0.353	1.38 (0.68–2.80)	0.366	1.08 (0.49–2.39)	0.857	1.12 (0.49–2.56)	0.791
Recessive model TT vs. CT+CC	1.22 (0.45–3.33)	0.695	1.25 (0.46–3.45)	0.662	0.80 (0.28–2.27)	0.675	0.90 (0.30–2.77)	0.860
Allele model T vs. C	1.05 (0.68–1.62)	0.825	1.21 (0.76–1.94)	0.426	1.21 (0.76–1.91)	0.426	1.20 (0.76–1.88)	0.439
miRNA 608 rs4919510								
GG	1.00 (ref.)				1.00 (ref.)			
GC	1.47 (0.53–4.05)	0.461	1.55 (0.55–4.36)	0.409	0.91 (0.28–3.04)	0.883	1.08 (0.31–3.78)	0.901
CC	1.01 (0.40–2.55)	0.977	1.02 (0.40–2.62)	0.962	1.42 (0.52–3.93)	0.497	1.58 (0.56–4.47)	0.389
Dominant model GC+CC vs. GG	1.45 (0.72–2.94)	0.303	1.52 (0.74–3.12)	0.225	0.70 (0.28–1.78)	0.456	0.76 (0.29–1.99)	0.580
Recessive model CC vs. GC+GG	1.14 (0.47–2.78)	0.772	1.17 (0.47–2.89)	0.738	1.25 (0.47–3.34)	0.652	1.43 (0.52–3.93)	0.485
Allele model C vs. G	0.99 (0.64–1.52)	0.964	1.23 (0.78–1.95)	0.378	1.20 (0.76–1.89)	0.428	1.05 (0.67–1.63)	0.839
miRNA 146A rs2910164								
CC	1.00 (ref.)				1.00 (ref.)			
CG	0.78 (0.25–2.44)	0.669	0.74 (0.23–2.37)	0.610	0.30 (0.07–1.28)	0.104	0.34 (0.08–1.45)	0.144
GG	0.94 (0.33–2.73)	0.914	0.97 (0.33–2.87)	0.951	0.42 (0.11–1.58)	0.200	0.46 (0.12–1.78)	0.261
Dominant model CG+GG vs. CC	0.82 (0.41–1.64)	0.575	0.76 (0.38–1.54)	0.446	0.63 (0.26–1.53)	0.310	0.65 (0.26–1.60)	0.345
Recessive model GG vs. CG+CC	0.88 (0.31–2.49)	0.814	0.88 (0.31–2.54)	0.814	0.38 (0.10–1.39)	0.144	0.42 (0.11–1.56)	0.194
Allele model G vs. C	0.77 (0.50–1.20)	0.246	1.16 (0.73–1.85)	0.535	0.89 (0.57–1.41)	0.630	0.83 (0.53–1.30)	0.414
miRNA 196A2 rs11614913								
CC	1.00 (ref.)				1.00 (ref.)			
CT	0.55 (0.23–1.31)	0.173	0.58 (0.24–1.43)	0.237	0.76 (0.22–2.64)	0.670	0.78 (0.22–2.80)	0.705
TT	0.61 (0.27–1.37)	0.227	0.67 (0.29–1.52)	0.334	1.73 (0.55–5.41)	0.348	1.85 (0.57–6.00)	0.309
Dominant model CT+CC vs. TT	0.76 (0.38–1.52)	0.443	0.77 (0.38–1.56)	0.461	0.50 (0.21–1.17)	0.110	0.49 (0.20–1.17)	0.109
Recessive model CC vs. CT+TT	0.58 (0.27–1.23)	0.157	0.63 (0.29–1.36)	0.240	1.28 (0.43–3.81)	0.664	1.33 (0.43–4.10)	0.620
Allele model T vs. C	1.00 (0.63–1.49)	0.876	0.76 (0.48–1.20)	0.239	0.74 (0.47–1.15)	0.181	0.96 (0.62–1.50)	0.863
miRNA 196A2 rs12304647								
AA	1.00 (ref.)				1.00 (ref.)			
AC	1.95 (0.22–17.37)	0.550	2.32 (0.25–21.50)	0.460	2.57 (0.25–26.04)	0.424	2.86 (0.28–29.42)	0.378
CC	2.93 (0.32–26.56)	0.340	3.01 (0.32–28.24)	0.335	1.83 (0.17–19.31)	0.617	1.95 (0.18–21.10)	0.582
Dominant model AC+CC vs. AA	0.72 (0.38–1.37)	0.311	0.83 (0.43–1.62)	0.583	1.49 (0.67–3.31)	0.333	1.56 (0.68–3.55)	0.291
Recessive model CC vs. AC+AA	2.29 (0.26–20.06)	0.455	2.60 (0.29–23.66)	0.397	2.28 (0.23–22.63)	0.483	2.50 (0.25–25.32)	0.437
Allele model C vs. A	1.46 (0.88–2.44)	0.146	0.95 (0.55–1.65)	0.856	0.85 (0.50–1.45)	0.539	1.50 (0.90–2.54)	0.128
miRNA 27a rs895819								
TT	1.00 (ref.)				1.00 (ref.)			
TC	0.67 (0.15–2.98)	0.600	0.64 (0.14–2.94)	0.570	0.74 (0.10–5.58)	0.766	0.88 (0.11–7.00)	0.903
CC	0.85 (0.19–3.92)	0.839	0.79 (0.17–3.74)	0.770	0.72 (0.09–5.60)	0.754	0.87 (0.11–7.17)	0.898
Dominant model TC+CC vs. TT	0.77 (0.40–1.47)	0.432	0.79 (0.41–1.52)	0.483	1.00 (0.46–2.16)	0.985	1.00 (0.45–2.21)	0.992
Recessive model CC vs. TC+TT	0.73 (0.17–3.19)	0.680	0.70 (0.16–3.11)	0.636	0.73 (0.10–5.38)	0.756	0.88 (0.11–6.80)	0.899
Allele model C vs. T	1.18 (0.72–1.96)	0.510	0.81 (0.47–1.40)	0.453	0.80 (0.47–1.37)	0.422	1.18 (0.70–1.97)	0.536

* OR_adj_ and *P*-value were calculated and adjusted for age, gender, alcohol consumption, and family history of tumor by a logistic regression model.

**Table 5:** Associations between the eight miRNA-SNPs and the risk of LC stratified by drinking

SNP/Genotype/Genetic model	Drinking				Non-drinking			
	OR (95%CI)	*P*	OR_adj_ (95%CI)^*^	*P*	OR (95%CI)	*P*	OR_adj_ (95%CI)^*^	*P*
miRNA 423 rs6505162								
CC	1.00 (ref.)				1.00 (ref.)			
CA	0.36 (0.04–3.52)	0.379	0.49 (0.05–5.14)	0.549	0.35 (0.11–1.15)	0.083	0.36 (0.11–1.22)	0.100
AA	0.14 (0.01–1.42)	0.096	0.20 (0.02–2.11)	0.179	0.38 (0.11–1.29)	0.120	0.39 (0.11–1.33)	0.131
Dominant model CA+AA vs.CC	1.95 (0.76–4.99)	0.164	1.99 (0.72–5.52)	0.187	0.82 (0.50–1.35)	0.436	0.84 (0.51–1.41)	0.512
Recessive model AA vs. CA+CC	0.24 (0.03–2.29)	0.216	0.32 (0.03–3.21)	0.333	0.36 (0.11–1.17)	0.090	0.37 (0.11–1.23)	0.105
Allele model A vs.C	1.24 (0.59–2.61)	0.564	1.27 (0.58–2.78)	0.550	0.78 (0.52–1.16)	0.218	0.79 (0.52–1.19)	0.264
miRNA 6811 rs2292879								
AA	1.00 (ref.)				1.00 (ref.)			
AG	2.87 (0.28–29.71)	0.377	3.19 (0.28–36.87)	0.353	2.72 (0.32–23.19)	0.361	3.74 (0.43–32.89)	0.235
GG	4.67 (0.42–52.12)	0.211	4.38 (0.36–53.36)	0.247	4.12 (0.48–35.37)	0.197	5.55 (0.63–49 .11)	0.123
Dominant model AG+GG vs.AA	0.77 (0.29–2.00)	0.584	0.90 (0.32–2.53)	0.835	0.70 (0.43–1.16)	0.168	0.73 (0.44–1.21)	0.224
Recessive model GG vs.AG+AA	3.38 (0.33–34.09)	0.303	3.61 (0.32–40.38)	0.297	3.26 (0.39–27.48)	0.277	4.47 (0.52–38.86)	0.174
Allele model G vs.A	0.99 (0.45–2.19)	0.978	1.14 (0.49–2.66)	0.757	0.84 (0.56–1.26)	0.399	0.88 (0.58–1.34)	0.544
miRNA 16-1 rs1022960								
CC	1.00 (ref.)				1.00 (ref.)			
CT	0.57 (0.16–2.07)	0.395	1.01 (0.24–4.26)	0.991	1.68 (0.74–3.83)	0.217	1.84 (0.79–4.26)	0.158
TT	0.71 (0.20–2.48)	0.593	0.96 (0.24–3.81)	0.956	1.29 (0.59–2.79)	0.525	1.41 (0.64–3.11)	0.396
Dominant model CT+TT vs.CC	0.72 (0.27–1.89)	0.505	1.04 (0.37–2.95)	0.946	1.37 (0.81–2.34)	0.244	1.40 (0.81–2.40)	0.231
Recessive model TT vs.CT+CC	0.64 (0.20–2.05)	0.455	0.98 (0.27–3.57)	0.978	1.42 (0.67–2.99)	0.361	1.52 (0.72–3.33)	0.259
Allele model T vs.C	0.74 (0.38–1.44)	0.371	1.00 (0.49–2.05)	0.998	1.25 (0.87–1.79)	0.220	1.29 (0.89–1.85)	0.177
miRNA 608 rs4919510								
GG	1.00 (ref.)				1.00 (ref.)			
GC	1.27 (0.28–5.87)	0.757	1.43 (0.29–7.18)	0.664	1.14 (0.52–2.48)	0.746	1.27 (0.57–2.80)	0.562
CC	3.25 (0.84–12.56)	0.088	3.36 (0.82–13.80)	0.092	0.80 (0.40–1.58)	0.520	0.81 (0.40–1.63)	0.554
Dominant model GC+CC vs.GG	0.51 (0.18–1.51)	0.224	0.57 (0.18–1.86)	0.351	1.35 (0.76–2.40)	0.302	1.49 (0.83–2.68)	0.183
Recessive model CC vs.GC+GG	2.44 (0.66–9.00)	0.179	2.60 (0.67–10.16)	0.170	0.89 (0.46–1.71)	0.721	0.92 (0.47–1.81)	0.814
Allele model C vs.G	0.99 (0.51–1.91)	0.978	1.07 (0.54–2.15)	0.843	1.08 (0.76–1.54)	0.664	1.13 (0.79–1.62)	0.504
miRNA 146A rs2910164								
CC	1.00 (ref.)				1.00 (ref.)			
CG	1.00 (0.20–5.00)	1.000	0.87 (0.16–4.75)	0.873	0.52 (0.21–1.30)	0.164	0.52 (0.20–1.33)	0.172
GG	0.77 (0.18–3.23)	0.715	0.44 (0.09–2.12)	0.304	0.72 (0.31–1.69)	0.454	0.71 (0.30–1.68)	0.431
Dominant model CG+GG vs.CC	1.25 (0.43–3.65)	0.683	1.68 (0.54–5.26)	0.370	0.69 (0.40–1.12)	0.189	0.71 (0.40–1.23)	0.221
Recessive model GG vs.CG+CC	0.83 (0.20–3.36)	0.789	0.56 (0.12–2.51)	0.447	0.65 (0.28–1.49)	0.307	0.64 (0.27–1.49)	0.300
Allele model G vs.C	1.04 (0.54–2.02)	0.900	1.08 (0.53–2.16)	0.839	0.79 (0.55–1.13)	0.188	0.79 (0.55–1.14)	0.215
miRNA 196A2 rs11614913								
CC	1.00 (ref.)				1.00 (ref.)			
CT	0.48 (0.13–1.86)	0.289	0.63 (0.15–2.67)	0.529	0.81 (0.40–1.64)	0.556	0.84 (0.41–1.73)	0.643
TT	1.00 (0.29–3.50)	1.000	1.34 (0.34–5.24)	0.672	0.84 (0.44–1.61)	0.594	0.80 (0.44–1.65)	0.631
Dominant model CT+CC vs.TT	0.48 (0.18–1.33)	0.157	0.51 (0.18–1.47)	0.212	0.92 (0.54–1.57)	0.751	0.95 (0.55–1.64)	0.842
Recessive model CC vs.CT+TT	0.75 (0.23–2.43)	0.632	0.99 (0.28–3.54)	0.984	0.83 (0.45–1.52)	0.541	0.85 (0.46–1.58)	0.603
Allele model T vs.C	0.66 (0.34–1.29)	0.224	0.73 (0.36–1.47)	0.381	0.90 (0.64–1.29)	0.576	0.92 (0.64–1.32)	0.653
miRNA 196A2 rs12304647								
AA	1.00 (ref.)				1.00 (ref.)			
AC	2.86 (0.28–29.75)	0.378	3.32 (0.30–37.26)	0.330	2.21 (0.46–0.78)	0.325	2.65 (0.53–13.28)	0.237
CC	4.50 (0.41–49.63)	0.219	4.28 (0.36–50.43)	0.248	2.12 (0.43–0.50)	0.358	2.34 (0.46–11.95)	0.308
Dominant model AC+CC vs.AA	0.78 (0.30–2.00)	0.598	0.94 (0.35–2.59)	0.911	1.11 (0.67–1.84)	0.694	1.21 (0.72–2.03)	0.474
Recessive model CC vs.AC+AA	3.38 (0.33–34.09)	0.303	3.67 (0.34–39.92)	0.285	2.18 (0.45–10.46)	0.332	2.52 (0.51–12.46)	0.257
Allele model C vs.A	0.99 (0.46–2.16)	0.987	1.15 (0.51–2.61)	0.741	1.15 (0.75–1.76)	0.521	1.23 (0.80–1.90)	0.346
miRNA 27a rs895819								
TT	1.00 (ref.)				1.00 (ref.)			
TC	1.25 (0.16–9.67)	0.831	2.67 (0.29–24.49)	0.384	0.67 (0.22–2.02)	0.473	0.70 (0.22–2.18)	0.534
CC	0.77 (0.09–6.45)	0.809	1.05 (0.11–10.23)	0.964	0.74 (0.24–2.30)	0.600	0.78 (0.24–2.51)	0.681
Dominant model TC+CC vs.TT	1.56 (0.60–4.08)	0.362	2.56 (0.85–7.72)	0.095	0.87 (0.53–1.44)	0.584	0.86 (0.52–1.44)	0.572
Recessive model CC vs. TC+TT	1.06 (0.14–7.97)	0.954	1.88 (0.22–16.18)	0.567	0.69 (0.23–2.06)	0.511	0.73 (0.24–2.23)	0.580
Allele model C vs.T	1.38 (0.62–3.05)	0.434	2.11 (0.86–5.15)	0.103	0.87 (0.57–1.30)	0.489	0.86 (0.57–1.31)	0.490

* OR_adj_ and *P*-value were calculated and adjusted for age, gender, smoking, and family history of tumor by a logistic regression model.

## Discussion

In the present study, we evaluated the association between eight polymorphisms in eight miRNAs and the risk of developing LC in a population from Southern Chinese. The study consisted of 132 LC cases and 214 cancer-free controls. Our findings primarily indicate that smoking individuals carrying CA or AA genotypes in miRNA-423 rs6505162 were less likely to develop LC.

rs6505162 (C>A) loci was in miRNA-423 gene, which localizes to the frequently amplified region of chromosome 17q11.2 and can produce two mature sequences: miRNA-423-3p and miRNA-423-5p [26,27]. Li et al. [28] reported that miRNA-423 rs6505162 polymorphism was associated with overall cancer susceptibility according to a meta-analysis of 35 eligible articles in 2020. Results of previous studies demonstrated that miRNA-423 plays different roles in the tumorigenesis and progression of several malignancies. Pourmoshir et al. [29] found that CC genotype of miRNA-423 rs6505162 exhibited a significant increased risk of breast cancer (BC) in women of Isfahan central province of Iran. However, Morales-Pison et al. [30] have proposed that it is the rs6505162-A allele that is responsible for the heightened risk of BC in South American women. Furthermore, they have highlighted that the rs6505162-A allele facilitates expression of mature miRNA-423 sequences (3p and 5p) in BC cell and that pre-miRNA-423-A expression induced cell proliferation, as well as enhance cell migration and invasion in BC [24]. It was also reported that miRNA-423 rs6505162 C>A might be associated with a significantly increased risk of esophageal cancer in smoking patients [31]. Results from two other studies [32,33] indicated that rs6505162 polymorphisms in miRNA-423 might be a protective factor against prostate cancer (PCa) for the overexpression of miRNA-423-5p in PCa cells can induce inhibition of glucose and amino acids metabolism as well as down-regulate the expression and activity of MALAT1, which can promote PCa cells proliferation, migration, and invasion. Moazeni-Roodi et al. [34] suggested that miRNA-423 rs6505162 variant significantly decreased the risk of cancer, including gastrointestinal cancer, colorectal cancer and LC among Asian population. Yet another study in Shanghai of China showed that miRNA-423 rs6505162 C > A polymorphisms were not associated with the risk of LC [35].

There have been limited published studies investigating the association between the miRNA-6811 rs2292879 polymorphism and human diseases. Wang et al. [36] conducted a study and did not find any significant association between this SNP and lung cancer. Nevertheless, our present study suggests that rs2292879 GG genotype might confer a positive impact on LC risk among Chinese Southern population. It is important to validate these findings in larger, well-designed studies, as the observed genotype frequencies for this SNP were not in accordant with the HWE in the control group.

We did not find any significant correlations between the remaining six miRNA SNPs (miRNA-16-1 rs1022960, miRNA-608 rs4919510, miRNA-146A rs2910164, miRNA-196A2 rs11614913, miRNA-196A2 rs12304647, and miRNA-27a rs895819) and LC risk. Our findings about the role of miRNA-16-1 rs1022960 and miRNA-608 rs4919510 in LC genetic susceptibility were consistent with the previous studies [37–39]. However, the associations between the remaining four miRNA SNPs and LC susceptibility are still controversial. Several studies suggested miRNA-146a2 rs2910164 polymorphism may play a role in increasing the risk of LC [40,41], but recently a study indicated that more evidence was needed to verify the relationship between rs2910164 SNPs and non-small cell lung cancer (NSCLC) susceptibility [42]. Qiu et al. found that miRNA-196a-2 rs11614913 was associated with a decreased risk of NSCLC in females [42]. But Hong et al. reported that polymorphisms in miRNA-196a2 rs11614913 might increase NSCLC risk [43], and Yin et al. proposed that individuals with both exposure to cooking oil fumes and risk genotypes of miRNA-196a2 rs11614913 were in a higher risk of LC [44]. Results of two separate studies showed that the miRNA-196A2 rs12304647 CC genotype exhibited a protective effect against the development of hepatocellular carcinoma in Malaysian and Korean patients with chronic hepatitis B virus infection and cirrhosis [45,46]. However, the existing body of research on the correlation between this SNP and the vulnerability to LC is currently limited. Extensive documentation has demonstrated the involvement of the miRNA-27a rs895819 polymorphism in susceptibility to various types of cancer. Nevertheless, only one single study has reported a higher risk of NSCLC in individuals bearing rs895819 G allele or GG and AG genotypes within Chinese population [47].

The aforementioned inconsistent findings, encompassing our own results and contentious published findings, may be attributed to variations in the study population, sample size, and tumor-specific etiology among different studies. However, it is important to acknowledge several limitations in this particular case–control study. First, the inclusion of cases and controls solely from the South of China may not provide a comprehensive representation of the general population. Second, due to the relatively small population size of Hainan province in China, the sample size could not be sufficiently large, potentially restricting the statistical power of our study. Finally, the present study did not evaluate the cancer pathological types, stages, and the interaction between SNPs and smoking or drinking.

In conclusion, the finding suggest that polymorphisms in miRNA-423 rs6505162 C>A may confer decreased individual susceptibility to LC among smokers from Southern China, whereas the GG genotype in miRNA-6811 rs2292879 may pose a risk for the population of Southern Chinese. However, further studies involving larger sample sizes, diverse ethnic populations, and functional analysis are necessary to validate or challenge the outcomes of this preliminary study.

## Data Availability

The datasets generated and/or analyzed during the current study are available from the corresponding author on reasonable request.

## Abbreviations

HWE: Hardy–Weinberg equilibrium
LC: lung cancer
MAF: minor allele frequency
ncRNA: non-coding small RNA
NSCLC: non-small cell lung cancer
OR: odds ratio
RISC: RNA-induced silencing complex
SNP: single-nucleotide polymorphism
95%CI: 95% confidence interval
